# Late Complications After European Medicines Agency-Approved Chimeric Antigen Receptor T-Cell Therapy in Hematological Malignancies: A Scoping Review

**DOI:** 10.3390/curroncol33080461

**Published:** 2026-08-01

**Authors:** Michael Eisenmann, Zhounan Zhu, Volker Arndt, Melissa S. Y. Thong

**Affiliations:** 1 Cancer Survivorship Outcomes and Epidemiology (C071), German Cancer Research Center (DKFZ), Im Neuenheimer Feld 280, 69120 Heidelberg, Germany; 2 Medical Faculty, University of Heidelberg, 69120 Heidelberg, Germany

**Keywords:** hematological malignancies, chimeric antigen receptor T-cell, late complications, non-relapse mortality

## Abstract

Chimeric antigen receptor T-cell (CAR-T) therapy has substantially improved outcomes for patients with B-cell-associated hematological malignancies. While acute and early toxicities are well characterized, late complications (LCs) remain poorly understood. This scoping review aimed to identify and map the existing evidence on LCs in adult patients with hematological malignancies treated with CAR-T products approved by the European Medicines Agency (EMA). PubMed, Web of Science, and the Cochrane Library were searched for primary studies published from 2018 onwards. We included studies of adult patients receiving EMA-approved CAR-T therapies targeting CD19 or CD269. LCs were defined as diagnosis-based adverse events occurring ≥12 months after CAR-T infusion. Of 7715 records identified, 261 studies underwent full-text screening and 18 met the inclusion criteria. LCs clustered mainly as infections and secondary malignancies (SMs). Non-relapse mortality was reported in seven studies, with infections and SMs frequently reported as causes when cause-of-death data were available. Future studies should place emphasis on long-term clinical events after CAR-T therapy to improve the management of LCs and ultimately support better long-term outcomes for patients.

## Simple Summary

People treated with chimeric antigen receptor T-cell (CAR-T) therapy may live longer, but some health problems can appear months or years after treatment. While early side effects of CAR-T therapy are well known, less is known about later complications and how they are reported. In this review, we examined published studies of adults with blood cancers who received CAR-T therapy approved by the European Medicines Agency and looked at complications reported at least one year after treatment. We focused on these products because they are relatively well established in clinical practice. Infections and new cancers were the most commonly reported problems and were also frequently reported causes of deaths not related to cancer relapse. However, studies used different follow-up periods and reporting methods, making results difficult to compare. Future studies should give more attention to long-term clinical events after CAR-T therapy, so that late complications can be better managed and patients can have better long-term outcomes.

## 1. Introduction

Chimeric antigen receptor T-cell (CAR-T) therapy involves collecting a patient’s T cells and genetically modifying them to recognize and attack tumor cells that express a specific surface antigen [1]. Since the first approval by the U.S. Food and Drug Administration in 2017 [2] and subsequent approval by the European Medicines Agency (EMA) in 2018 of axicabtagene ciloleucel and tisagenlecleucel [3], a total of six CAR-T products (axicabtagene ciloleucel, brexucabtagene autoleucel, ciltacabtagene autoleucel, idecabtagene vicleucel, lisocabtagene maraleucel and tisagenlecleucel) have become available in Europe [4]. Four of these products target CD19 and are used to treat B-cell hematological malignancies, including large B-cell lymphoma. The other two target CD269-positive plasma cells and are used to treat multiple myeloma [4]. Hematological malignancies have historically been associated with poor outcomes. For example, patients with refractory diffuse large B-cell lymphoma (DLBCL) showed a complete response rate of only 7%, a median overall survival of 6.3 months, and an approximately 20% survival rate at 2 years [5]. Against this background, early studies of CD19-directed CAR-T therapy in refractory aggressive B-cell lymphoma, including DLBCL, demonstrated substantially higher response rates, with complete responses observed in 57% of patients (4/7), of whom 3 patients had ongoing complete responses lasting at least 12 months [6].

Despite these therapeutic successes, CAR-T therapy is associated with a broad spectrum of adverse events [7,8]. Acute toxicities, particularly cytokine release syndrome (CRS) and immune effector cell-associated neurotoxicity syndrome (ICANS), usually occur shortly after infusion and are now relatively well characterized [9]. Currently, with advances in treatment strategies, patients are surviving long enough for late complications (LCs) to become clinically relevant [10]. Reported LCs include cytopenias, hypogammaglobulinemia, infections, neurologic and cardiovascular events, and secondary malignancies (SMs) [11]. These complications can have serious consequences. Treatment-related adverse events contribute to non-relapse mortality (NRM), and infections account for approximately half of reported NRM events [12]. Some complications begin soon after treatment and persist, whereas others first appear later in the follow-up period [13]. However, the current evidence base on LCs remains limited by relatively short follow-up. Some studies assessing complications after CAR-T therapy have captured events only within the first 3 or 6 months [14,15]. Such follow-up may be insufficient to characterize complications with longer latency. Although several recent cohorts have begun to report adverse events beyond 1 year, long-term evidence remains relatively sparse [11,16]. Beyond the spectrum of events themselves, the way LCs are captured and reported remains a challenge. To date, no standardized clinical guidelines specify the appropriate duration of follow-up after CAR-T therapy, creating a risk of underreporting late adverse events, particularly SMs, which have recently drawn explicit regulatory attention [17]. Together, these considerations underscore the need for a temporally explicit framework when characterizing the long-term safety profile of CAR-T therapy.

Therefore, this scoping review aims to systematically map the existing evidence on LCs after CAR-T therapy. For this review, LCs are defined as adverse events with first onset one year or later after CAR-T infusion. By summarizing which LCs have been reported and how they have been described, this review aims to provide a comprehensive overview of long-term LCs after CAR-T therapy and may help inform future patient management and the design of subsequent research studies.

## 2. Methods

### 2.1. Literature Search

We conducted a scoping review in accordance with the methodological guidance provided in the Joanna Briggs Institute (JBI) Manual for Evidence Synthesis [18], with the aim of mapping and characterizing LCs after CAR-T therapy. Findings were reported according to the PRISMA Extension for Scoping Reviews (PRISMA-ScR) [19]. The study protocol was registered with the Open Science Framework (DOI: 10.17605/OSF.IO/7UTFS).

We searched PubMed, the Cochrane Library, and Web of Science for studies published from 1 January 2018 to 28 June 2025. The search terms were developed with the support of a specialist librarian. The complete search strategies for each database are provided in Table S1.

### 2.2. Inclusion and Exclusion Criteria

Eligible studies were peer-reviewed English-language publications from 2018 onward that reported CAR-T therapy for hematological malignancies and described at least one clinically relevant, diagnosis-based LC. The search strategy was oriented toward EMA-approved CAR-T products targeting CD19 or CD269. Studies were eligible when they provided sufficient clinical context to identify the treated population, CAR-T product, follow-up duration, and timing or attribution of the reported complication. We limited the review to EMA-approved CAR-T products to maintain a clear, clinically relevant scope and to focus on therapies with more established clinical use and longer follow-up data.

Studies were excluded if they focused on pediatric populations, non-hematological malignancies such as solid tumors, preclinical or experimental models, or therapies outside the predefined CAR-T product scope. We also excluded reviews, commentaries, editorials, conference abstracts or posters, dissertations, and publications that reported only acute or early adverse events occurring before 12 months after CAR-T infusion, nonspecific symptoms, laboratory findings without diagnostic context, or adverse events for which occurrence at 12 months or later after CAR-T infusion could not be determined.

### 2.3. Data Extraction

Two authors (ME, ZZ) independently extracted and charted data from the selected studies using the structure provided by Covidence. Disagreements between the reviewers were resolved by discussion with the senior author (MT) when necessary. Extracted and charted variables included author, year, study design, country, CAR-T product, treated disease, number of patients, age of patients, median follow-up, comparator or patient denominator, reported LCs, LC cluster, LC time point, NRM count or rate, NRM time point, and cause of NRM.

### 2.4. Assessment of LCs

LCs were screened for an indication that onset occurred at least 12 months after CAR-T infusion. Where studies presented cumulative adverse-event tables covering the entire observation period, individual events were separated and retained as LCs only when their reported timing met this threshold. LCs were additionally captured from narrative descriptions within the main text, where they were reported as incidental findings rather than as predefined outcomes. Extracted LCs were assigned to one of twelve predefined clusters. These clusters were developed based on prior publications on CAR-T toxicities and two recent review articles [7,8]. The clusters comprised cardiopulmonary and vascular complications, dermatologic complications, endocrine or metabolic complications, gastrointestinal complications, general or systemic conditions, hematological or immunologic complications, infectious complications, musculoskeletal complications, neurologic complications, SMs, sexuality or fertility-related complications, and “other” category for complications that were unspecified or did not fit into the predefined clusters.

### 2.5. Assessment of Study Quality

We assessed the quality of the included cohort studies using the JBI Critical Appraisal Checklist for Cohort Studies [20]. As the purpose of this scoping review was to describe the available evidence, studies were not excluded on the basis of their appraisal results. Each checklist item was rated as yes, no, unclear, or not applicable. Early-phase and single-arm interventional studies were not assessed using the JBI cohort checklist because several core domains of this instrument, including comparison-group selection, exposure measurement across groups, and control of confounding, were not applicable to uncontrolled study designs.

## 3. Results

### 3.1. Study Selection

**Figure 1:**
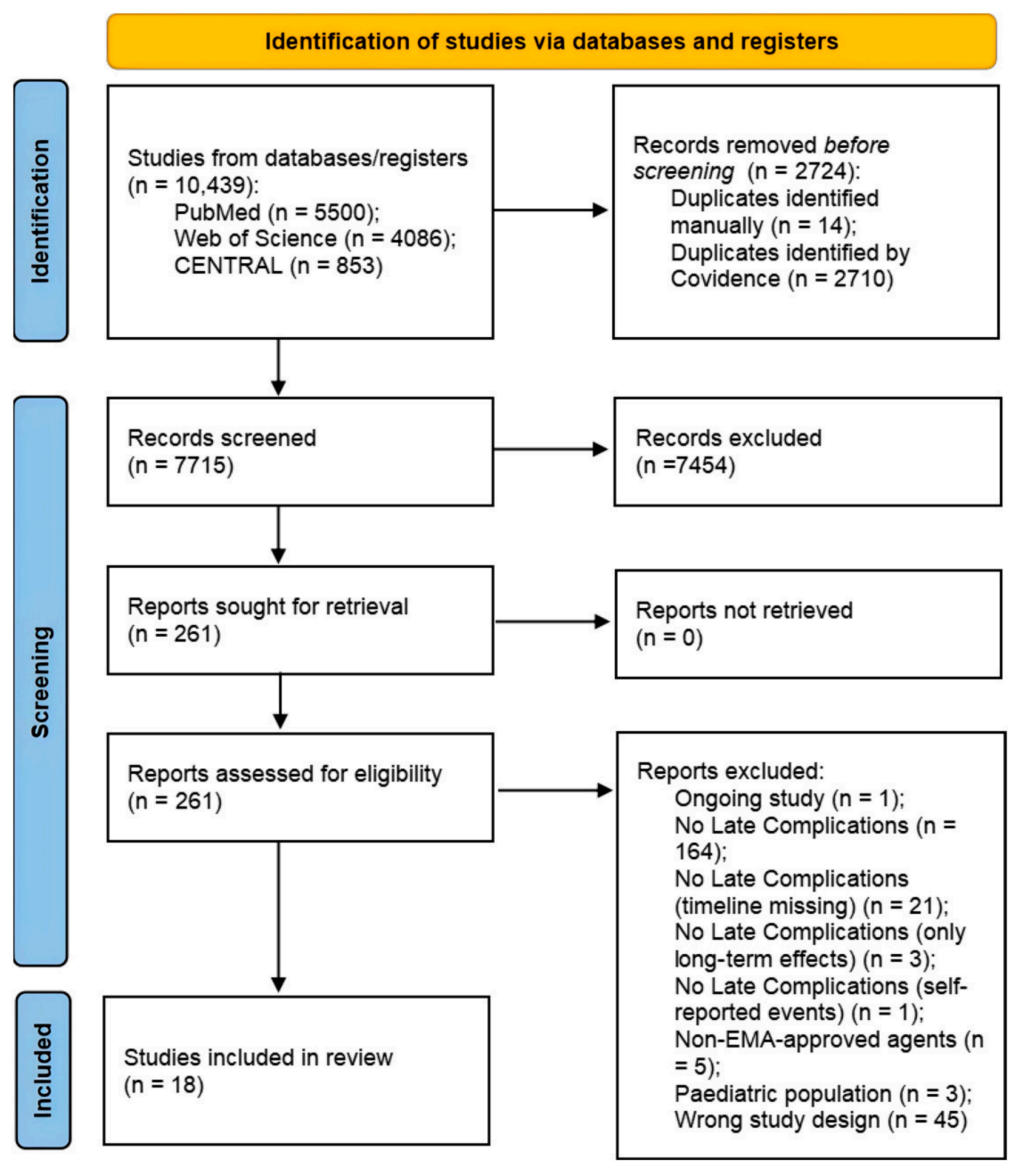
Study selection (PRISMA flowchart).

A total of 10,439 articles were retrieved and 2724 duplicates were removed, leaving 7715 articles for screening by title, abstract and full text. Thereafter, 18 studies met the inclusion criteria and were selected for data extraction (Figure 1).

### 3.2. Study Characteristics

The 18 included studies were published between 2019 and 2025. The median publication year was 2024, and 13 studies were published from 2023 onward. The evidence base consisted of nine cohort studies, eight single-arm or early-phase clinical trials, and one randomized phase 3 trial. Study size varied considerably: five studies included more than 300 patients, six included 100 to 300 patients, and seven included fewer than 100 patients. Overall, 3036 patients received CAR-T therapy across the included studies.

Most studies originated from the United States (*n* = 15), followed by France (*n* = 3), Israel (*n* = 2), and Germany (*n* = 2). Additional individual studies included sites in Canada, the Netherlands, Spain, and Switzerland, reflecting a predominantly North American and European evidence base.

Axicabtagene ciloleucel was the most frequently reported CAR-T product (*n* = 13), followed by tisagenlecleucel (*n* = 7), brexucabtagene autoleucel (*n* = 5), lisocabtagene maraleucel (*n* = 5), and ciltacabtagene autoleucel (*n* = 1). Idecabtagene vicleucel was not represented. The underlying diseases were dominated by B-cell malignancies. Most studies included patients with diffuse large B-cell lymphoma (DLBCL; *n* = 14), followed by primary mediastinal B-cell lymphoma (PMBCL; *n* = 10), transformed follicular lymphoma (tFL; *n* = 8), and follicular lymphoma (FL; *n* = 4).

Eleven studies reported a study-level median or mean age of 60 years or older, six reported a median or mean age below 60 years, and one did not report age in the extracted text. Median follow-up exceeded 36 months in six studies, ranged from 24 to 36 months in four studies, and was less than 24 months in seven studies; one study reported follow-up only as up to 24 months rather than as a median follow-up estimate (Table 1).

### 3.3. Spectrum of LCs

Among the 3036 patients treated with CAR-T therapy across the included studies, 220 experienced at least one LC as defined in this review, with 21 patients experiencing more than one distinct LC (Table 2). In total, 241 LC events were reported. These included 43 unique LCs, while 11 events were not further characterized.

Infections were the most common specific LC category, affecting approximately 83 patients and accounting for 96 reported events. More than half of all infectious events remained unspecified (*n* = 59; 61.5%). Among specific reported LCs, myelodysplastic syndrome (*n* = 27) and pneumonia (*n* = 18) were the most frequently identified individual complications.

### 3.4. Cluster Allocation

All extracted LCs could be allocated to seven of the 12 predefined clusters: neurologic, hematological/immunologic, infectious, cardiopulmonary and vascular, general or systemic conditions, SM, and other events. No LCs were reported in the predefined clusters for gastrointestinal, dermatologic, musculoskeletal, endocrine/metabolic, or sexual/fertility-related events. Dermatologic malignancies, including melanoma, nonmelanoma skin cancer, and skin squamous cell carcinoma, were categorized under secondary malignancy rather than under dermatologic events.

Infections and SMs were the most frequently reported clusters. Infectious LCs accounted for 16 types of manifestation, including 13 specific and three unspecific manifestations, and were reported in 14 of 18 studies. SMs accounted for 24 types of manifestation, including 21 specific and three unspecific manifestations, and were reported in 10 of 18 studies. An overview of all types of LCs by cluster is provided in Table S2. The infectious cluster included bacterial, viral, fungal, and opportunistic infections, such as bacteremia, bacterial sinusitis, candidemia, COVID-19, viral encephalitis, hepatitis B reactivation, herpes zoster, Pneumocystis jirovecii pneumonia, pneumonia, respiratory viral infection, urinary tract infection, and bacterial sepsis, as well as several unspecified infectious events. The SM cluster was the most diverse cluster by event type and included hematologic malignancies, therapy-related myeloid neoplasms, skin cancers, solid tumors, and several unspecified second primary malignancies. Less frequently represented clusters included neurologic events, hematological or immunologic events, cardiopulmonary or vascular events, general or systemic conditions, and other unspecified causes. The hematologic or immunologic cluster included neutropenia, thrombocytopenia, anemia, febrile neutropenia, graft-versus-host disease, and hepatic vein thrombosis. Neurologic entries included unspecified neurologic or psychiatric disorders and suicide, whereas cardiopulmonary or vascular entries included cardiopulmonary arrest, unspecified cardiovascular events, hypoxia with acute respiratory distress syndrome and hypotension, and intracranial hemorrhage.

### 3.5. Timing of LCs

The timing of LCs varied substantially across studies and was not reported uniformly. Several studies reported LCs within defined follow-up windows, such as 12–24 months, 24–48 months, or 12–60 months after infusion, rather than providing exact onset dates for each complication. When exact timings were available, late infectious events were reported at approximately 12–22 months after infusion, including Pneumocystis jirovecii pneumonia, bacterial sepsis, COVID-19, hepatitis B reactivation, and sepsis. SMs were generally reported later, ranging from approximately 13 to 60 months after CAR-T therapy, with some malignancy-related events occurring beyond 40 months. A small number of very late complications were reported after 60 months, mainly in long-term follow-up cohorts.

### 3.6. Non-Relapse Mortality and Causes of Death

NRM or an equivalent non-progression mortality endpoint was reported in 7 of 18 studies. The definition and reporting of NRM varied across studies, including explicitly defined NRM, death without relapse or progression, and non-progressive disease (PD) mortality. Reported NRM rates ranged from 1.7% at 1 year [35] to 17.4% overall [25], with one study reporting 36-month non-PD mortality of 19% [23]. In studies with longer follow-up, cumulative NRM increased over time, reaching 14.0% at 4 years in one multicenter cohort [21]. The most frequently reported causes of NRM were infections and SMs. Infection-related NRM included COVID-19 pneumonia, bacterial sepsis, pneumonia, Pneumocystis jirovecii pneumonia, and severe influenza A pneumonia. Secondary malignancy-related NRM included therapy-related myeloid neoplasms, myelodysplastic syndrome, and acute myeloid leukemia. Eleven studies reported causes of death or fatal adverse events without formally defining them as NRM.

### 3.7. Results of the Study Quality Assessment

**Figure 2:**
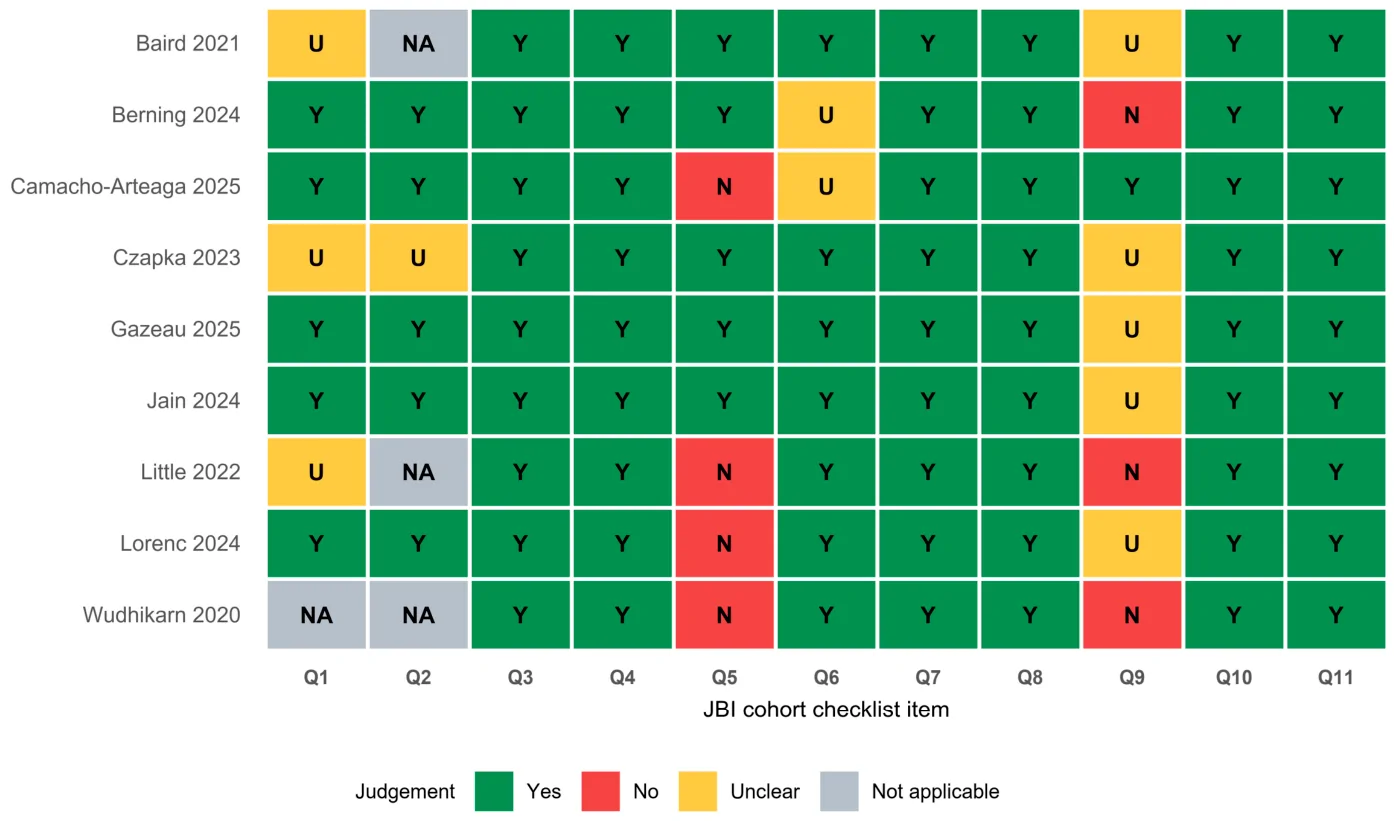
Quality appraisal of included cohort studies using the JBI cohort checklist. Studies shown in the figure include Baird 2021 [33], Berning 2024 [25], Camacho-Arteaga 2025 [10], Czapka 2023 [29], Gazeau 2025 [21], Jain 2024 [16], Little 2022 [32], Lorenc 2024 [27], and Wudhikarn 2020 [35].

Quality appraisal using the JBI cohort checklist was performed only for the nine cohort studies. Most applicable items were rated positively. Overall, 77 of 99 item-level judgments were rated as “Yes”, 11 as “Unclear”, 7 as “No” and 4 as “Not applicable”. The main methodological concerns related to whether the comparison groups were similar and recruited from the same population (Q1), whether exposures were measured similarly across groups (Q2), whether strategies to address confounding were clearly stated (Q5), whether participants were free of the outcome at the start of the study (Q6), and whether follow-up was complete or reasons for loss to follow-up were described and explored (Q9). (Figure 2 and Table S3).

## 4. Discussion

In this scoping review, we described LCs occurring one year or later after CAR-T therapy. Across 18 included studies, a total of 3036 patients received CAR-T therapy, of whom at least 220 experienced one or more LCs. Overall, we identified 241 LC events. The most frequently reported LC clusters were infectious complications and SMs. Other reported LCs included hematologic or immunologic complications, neurologic events, cardiopulmonary or vascular complications, and general or unspecified conditions. NRM was reported in several studies, and when causes were specified, fatal events were most often related to infections and SMs. Overall, these findings provide an overview of long-term LCs and NRM after CAR-T therapy.

LCs encompassed a broad clinical spectrum, among which infections and SMs were the most frequently reported LCs. Pneumonia and sepsis were the predominant infectious complications. Infections have traditionally been discussed mainly as early complications after CAR-T therapy, with previous studies showing that infectious events are particularly common within the first 6 months after infusion and peak during the early post-treatment period [15,37]. Our findings extend this perspective by showing that infections remain a prominent component of the late complication spectrum beyond 12 months. This suggests that infection risk after CAR-T therapy should not be viewed solely as an early post-infusion toxicity, but also as a relevant long-term survivorship issue [38].

SMs represented a second major LC. Unlike infections, which may occur throughout follow-up and are more linked to potentially preventable morbidity and mortality, SMs often require longer observation periods. Additionally, SMs are challenging to attribute solely to CAR-T therapy, as prior treatments, underlying disease biology, and age-related background risks may also contribute to their development [39]. Recent long-term follow-up data have similarly identified late infections and subsequent malignant neoplasms as key survivorship issues after CAR-T therapy [16], while a systematic review and meta-analysis has highlighted second primary malignancies as clinically relevant long-term adverse events in patients treated with CAR-T therapy [40]. Together, these findings indicate that long-term follow-up after CAR-T therapy should address both persistent infectious vulnerability and malignancy surveillance, rather than focusing only on acute toxicity or disease relapse.

The hematologic/immunologic cluster included neutropenia, thrombocytopenia, anemia, febrile neutropenia, graft-versus-host disease, and hepatic vein thrombosis. Recent studies on hematotoxicity associated CAR-T therapy have emphasized that prolonged or late cytopenias are not merely laboratory abnormalities, but may contribute to clinically relevant outcomes, particularly infection risk [41,42]. Although hematologic/immunologic LCs were less frequently reported than infections or SMs in the present review, they may lie upstream of several subsequent complications, especially infection-related morbidity and NRM. Therefore, future studies should capture cytopenias in greater detail rather than recording them only as present or absent, including their onset, duration, severity grade and recovery pattern. The neurologic cluster included unspecified neurologic disorders, unspecified psychiatric disorders, suicide, and unspecified causes. This low level of diagnostic detail contrasts with the detailed attention typically given to acute ICANS, suggesting that later neurocognitive, psychiatric, and patient-reported outcomes may be undermeasured. Long-term survivor studies have shown that neuropsychiatric symptoms can remain relevant after CAR-T therapy, even when they are not captured as classic acute neurotoxicity [43]. Similarly, the cardiopulmonary and vascular cluster included cardiopulmonary arrest, unspecified cardiovascular event, hypoxia with acute respiratory distress syndrome and hypotension, and intracranial hemorrhage. Cardiovascular complications after CAR-T therapy are often discussed in relation to early CRS-associated stress, but emerging cardio-oncology literature suggests that structured cardiac event definitions and longer follow-up are needed to understand the full burden of cardiopulmonary toxicity [44,45]. The general/systemic and other clusters, including pyrexia, missing causes, and unspecified causes, further illustrate the limitations of the source literature. These entries are difficult to interpret clinically but are useful methodologically: they show where reporting systems failed to provide enough diagnostic resolution for meaningful synthesis.

In addition, the assessment of LC severity remains challenging. Unlike acute toxicities related to CAR-T therapy such as CRS and ICANS, for which the American Society for Transplantation and Cellular Therapy (ASTCT) consensus grading system has provided standardized definitions and severity categories, LCs after CAR-T therapy encompass a much broader and more heterogeneous spectrum of conditions [46]. These events may involve hematological, infectious, neurologic, immune-mediated, endocrine, pulmonary, renal, cardiovascular, and secondary malignant complications, each of which may require different clinical grading systems or organ-specific severity criteria. Therefore, a single standardized framework is unlikely to capture the full clinical complexity of LCs across organ systems. This issue is further complicated by the inconsistent way severity is reported in the existing literature. Some studies report only the occurrence of LCs without grading their severity, whereas others use non-uniform thresholds, such as need for hospitalization, requirement for intervention, persistence beyond a certain time point, or attribution to CAR-T therapy [10,13,47]. Moreover, several LCs, such as prolonged cytopenia, hypogammaglobulinemia, recurrent infections, delayed neurotoxicity, and SMs, may evolve over time rather than present as discrete events with a clearly defined onset and grade. As a result, severity could not be reliably assigned to each individual LC, and graded comparisons across studies or clusters were not feasible. Therefore, the present synthesis focuses on the range and distribution of LCs rather than their graded clinical severity.

NRM represents an important outcome after CAR-T therapy. In this review, NRM was reported in 7 of 18 studies. Infections and SMs repeatedly appeared among reported causes of NRM. This is supported by a recent meta-analysis of NRM after CAR-T therapy, in which infections accounted for more than half of reported non-relapse deaths, followed by other malignancies. Notably, infections were identified as the predominant cause of NRM during the first year after CAR-T therapy, whereas the temporal patterns of SM development were not specifically evaluated [12]. Our findings complement these observations by characterizing the LC landscape beyond 12 months after CAR-T therapy, thereby offering additional insight into potential long-term NRM patterns among survivors. Although the available evidence does not establish that LCs directly caused NRM, it suggests that LC-associated mortality may be incompletely captured. If validated in prospective studies, systematic monitoring and reporting of infections, SMs, and cause-specific mortality may help clarify patterns of late NRM after CAR-T therapy and identify potentially modifiable contributors to long-term mortality [48]. Similar to LCs, NRM after CAR-T therapy is not yet reported within a universally harmonized framework. This issue is important because NRM is a competing-risk outcome: deaths unrelated to relapse or progression should be analyzed with relapse/progression as a competing event rather than simply summarized as aggregate mortality [49]. Experience from the hematopoietic cell transplantation field has shown that standardized definitions and adjudication algorithms can reduce inconsistency in cause-specific mortality assignment [50]. However, CAR-T-specific standards for reporting NRM remain limited, particularly regarding time windows, attribution, and cause of death. More consistent NRM reporting is needed to clarify how LCs may contribute to late mortality after CAR-T therapy.

The limited number of reported LC and NRM events also affected what could be inferred from the available evidence. Although this scoping review identified a broad range of LCs, most individual events were reported only rarely, and many appeared in single studies or as isolated cases. This limited event volume made it difficult to identify consistent patterns in the type, timing, or sequence of LCs after CAR-T therapy [7,11,13]. Infections and SMs were the most frequently reported clusters, but the available data were insufficient to determine whether specific infections, malignancy subtypes, or other organ-system complications follow reproducible temporal trajectories after infusion [10,12]. Similarly, although NRM was reported in several studies and was often linked to infections or SMs when causes were specified, the number of cause-specific NRM events was too small and inconsistently reported to support firm conclusions about late mortality patterns [49]. The interpretation of timing was also limited by heterogeneous reporting across studies. Some reports provided exact onset dates, whereas others used broad follow-up windows or cumulative adverse-event summaries [40]. As a result, it was often difficult to determine whether an event truly emerged late, persisted from an earlier phase, or was detected late because of longer surveillance. This is relevant for cytopenias, infections, and SMs, which may develop gradually. Therefore, the present scoping review could map the reported spectrum of LCs and NRM, but it could not establish reliable temporal patterns or identify clear event trajectories.

Of the 18 included studies, only nine had cohort designs and could be assessed using the JBI cohort checklist; most of the remaining studies were phase 1 or phase 2 clinical trials. Therefore, the JBI appraisal offers only a general indication of the quality of a selection of the included studies and should not be interpreted as an assessment of the entire evidence base. Although the included cohort studies generally showed acceptable quality, several concerns may affect the reliability and comparability of the reported LCs [20]. The main concerns were that comparison groups were not comparable or drawn from the same population, exposure was not consistently measured across groups, confounding factors were insufficiently considered, baseline outcome status was sometimes unclear, and follow-up was incomplete or losses to follow-up were not adequately addressed. Some comparison groups included patients with different baseline characteristics across groups. Consequently, differences in the reported LCs may have reflected differences between the groups or in data collection, rather than effects of CAR-T therapy itself [7]. In addition, limited reporting of confounding factors made it difficult to determine whether the reported complications were related to CAR-T therapy or to other factors, such as patients’ disease response to CAR-T therapy, immune reconstitution, prior or subsequent treatments, relapse, and supportive care [7,51]. When baseline outcome status was unclear, pre-existing conditions or ongoing toxicities may have been reported as newly developed LCs [52]. Incomplete follow-up may have resulted in LCs being missed, particularly among patients who died or were lost to follow-up. Differences in outcome definitions, follow-up periods, and reporting methods further limited comparisons across studies. Future studies should include appropriate comparison groups, define long-term safety outcomes, account for relevant confounding factors, and ensure longer and more complete follow-up to improve the reliability and comparability of the evidence.

A major strength of this scoping review is that, to the best of our knowledge, it is the first to systematically map LCs and NRM occurring 12 months or later after treatment with the six EMA-approved CAR-T products for hematological malignancies. This focus allowed LCs to be distinguished from acute and early toxicities and provided a structured overview of long-term safety outcomes across currently approved CAR-T therapy. This review has several limitations. First, few included studies used appropriate comparison cohorts, as most were designed to describe outcomes after CAR-T therapy rather than compare LCs with background risks in similar non-CAR-T populations. This limits causal inference and increases the risk of selection and attribution bias, particularly for secondary malignancies, where prior therapies, age-related morbidity, and background cancer risk must be considered. Second, because this scoping review focused on complications occurring more than 12 months after CAR-T therapy, complications arising within the first 12 months were not systematically extracted or analyzed. Moreover, the included studies generally lacked patient-level longitudinal data linking early and late complications within the same individuals. Consequently, we could not determine whether prior early complications were associated with an increased risk of subsequent late complications. Future longitudinal studies with patient-level follow-up are needed to clarify the temporal relationship between early and late complications. Third, not all EMA-approved CAR-T products were represented. Idecabtagene vicleucel was not reported in any included study, limiting the generalizability of the findings across currently approved CAR-T therapy and suggesting that LCs after less mature products may remain undercaptured. Fourth, the included studies were geographically skewed toward the United States, with fewer studies from European or other healthcare settings. Differences in follow-up structures, survivorship care, reporting standards, and regulatory frameworks may therefore influence the detection and documentation of LCs.

## 5. Conclusions

In conclusion, this scoping review highlights LCs reported at least 12 months after treatment with EMA-approved CAR-T products for hematological malignancies. Infections and SMs were among the most frequently reported LC categories. NRM was reported in several studies, and infections and SMs were frequently reported as causes when cause-of-death data were available. Future studies should place emphasis on long-term clinical events after CAR-T therapy to improve the management of LCs and ultimately support better long-term outcomes for patients.

## Figures and Tables

**Table 1:** Characteristics of included CAR-T therapy studies.

Author	Year	Study Design	Country	CAR-T Product	Treated Disease	No. of Patients	Age of Patients (Median)	Median Follow-Up
Camacho-Arteaga et al. [10]	2025	Prospective multicenter observational cohort	Spain	Axicabtagene ciloleucel; tisagenlecleucel	Aggressive LBCL (DLBCL, PMBCL, tFL)	391 treated; 172 included at the 3-month landmark	Median 60 years (axi-cel) and 66 years (tisa-cel); overall mean 58.5 years	13.9 months (IQR, 8.2–23.8)
Gazeau et al. [21]	2025	Retrospective multicenter cohort	France	Tisagenlecleucel; axicabtagene ciloleucel; brexucabtagene autoleucel; lisocabtagene maraleucel	B-NHL (DLBCL, tiBCL, MCL, FL, MZL, PMBCL)	539	63 years	25 months (IQR, 24–29)
Jagannath et al. [22]	2025	Phase Ib/II multicenter open-label trial (CARTITUDE-1)	United States	Ciltacabtagene autoleucel	Multiple myeloma	97	61 years	61.3 months
Shah et al. [23]	2025	Phase 1/2 multicenter single-arm trial (ZUMA-3 LTFU)	United States	Brexucabtagene autoleucel	B-cell ALL	78	42.5 years	41.6 months (IQR, 32.7–70.3)
Abramson et al. [24]	2024	Multicenter seamless-design study (TRANSCEND NHL 001)	United States	Lisocabtagene maraleucel	DLBCL, HGBCL, PMBCL, FL	345 leukapheresed; 270 liso-cel treated	63 years	24-month follow-up; survival follow-up median 29.3 months
Berning et al. [25]	2024	Retrospective multicenter study	Germany and Switzerland	Tisagenlecleucel; axicabtagene ciloleucel	DLBCL	172	61 years (<70 group) and 74 years (≥70 group)	8.15 months (range, 0.1–44.3) and 8.58 months (range, 0.4–42.3)
Jain et al. [16]	2024	Retrospective multicenter registry cohort	United States	Axicabtagene ciloleucel	DLBCL, PMBCL, tFL	298 leukapheresed; 275 treated	60 years	58 months (range, 0.16–68.7)
Locke et al. [26]	2024	Phase 1/2 multicenter single-arm cohort (ZUMA-1 Cohort 3)	United States, Canada, France, Germany, Israel, the Netherlands	Axicabtagene ciloleucel	DLBCL, PMBCL, tFL	38	51 years	31.9 months (range, 24.0–36.0) and 55.9 months (range, 48.0–60.0)
Lorenc et al. [27]	2024	Retrospective multicenter study	United States	Tisagenlecleucel; axicabtagene ciloleucel; brexucabtagene autoleucel; lisocabtagene maraleucel	DLBCL, HGBCL, MCL, FL	355	65 years	19.26 months (IQR, 7.5–33.2)
Neelapu et al. [28]	2024	Phase 2 multicenter single-arm trial (ZUMA-5 LTFU)	United States and France	Axicabtagene ciloleucel	FL, MZL, DLBCL	159 enrolled; 152 treated	60 years (FL) and 64 years (MZL)	41.7 months (range, 32.7–57.4) and 31.8 months (range, 8.3–52.3)
Czapka et al. [29]	2023	Single-center cohort	United States	Tisagenlecleucel; axicabtagene ciloleucel; brexucabtagene autoleucel; lisocabtagene maraleucel	DLBCL, HGBCL, tFL, MCL, PMBCL, ALL	73	64 years	Up to 24 months
Neelapu et al. [30]	2023	Phase 1/2 multicenter single-arm trial (ZUMA-1 5-year LTFU)	United States and Israel	Axicabtagene ciloleucel	DLBCL, PMBCL, tFL	111 enrolled; 101 treated	58 years	63.1 months (range, 58.9–68.4)
Westin et al. [31]	2023	Phase 3 randomized trial (ZUMA-7)	Multinational; article reports US and global trial sites	Axicabtagene ciloleucel	Large B-cell lymphoma	359 randomized; 180 assigned to axi-cel; 170 treated in safety set	Not reported	47.2 months (range, 39.8–60.0)
Little et al. [32]	2022	Retrospective single-center cohort	United States	Tisagenlecleucel; axicabtagene ciloleucel; brexucabtagene autoleucel; lisocabtagene maraleucel	DLBCL, tFL, HGBCL, MZL, PMBCL, FL, MCL, CLL, NHL	280	64 years	8.5 months (range, 0.2–44.0)
Baird et al. [33]	2021	Single-center cohort	United States	Axicabtagene ciloleucel	DLBCL, PMBCL, tFL	41	56 years	19.8 months
Shah et al. [34]	2021	Phase 1/2 multicenter single-arm open-label trial (ZUMA-3)	United States	Brexucabtagene autoleucel/KTE-X19	B-cell ALL	54 enrolled; 45 treated	46 years	22.1 months (range, 7.1–36.1)
Wudhikarn et al. [35]	2020	Retrospective single-center cohort	United States	Tisagenlecleucel; axicabtagene ciloleucel	DLBCL, transformed indolent B-cell lymphoma	60	63 years	9 months
Locke et al. [36]	2019	Phase 1/2 multicenter single-arm trial (ZUMA-1)	United States and Israel	Axicabtagene ciloleucel	DLBCL, PMBCL, tFL	119 enrolled; 108 treated	59 years (phase 1) and 58 years (phase 2)	27.1 months (range, 25.7–28.8)

Abbreviations: ALL, acute lymphoblastic leukemia; axi-cel, axicabtagene ciloleucel; B-NHL, B-cell non-Hodgkin lymphoma; CAR-T, chimeric antigen receptor T-cell; CLL, chronic lymphocytic leukemia; DLBCL, diffuse large B-cell lymphoma; FL, follicular lymphoma; HGBCL, high-grade B-cell lymphoma; IQR, interquartile range; KTE-X19, brexucabtagene autoleucel; LBCL, large B-cell lymphoma; liso-cel, lisocabtagene maraleucel; LTFU, long-term follow-up; MCL, mantle cell lymphoma; MZL, marginal zone lymphoma; NHL, non-Hodgkin lymphoma; PMBCL, primary mediastinal B-cell lymphoma; tFL, transformed follicular lymphoma; tiBCL, transformed indolent B-cell lymphoma; tisa-cel, tisagenlecleucel; ZUMA, a series of clinical trials evaluating axicabtagene ciloleucel or brexucabtagene autoleucel.

**Table 2:** Late complications reported after CAR-T therapy.

Author	Comparator/No. of Patients	LCs	Cluster	LCs Time Point	NRM Count/Rate	NRM Time Point	Cause of NRM
Camacho-Arteaga et al. [10]	Product comparison: axicabtagene ciloleucel *n* = 117 vs. tisagenlecleucel *n* = 55.	(1) Infections: 31 patients/44 events; (2) Neutropenia: 11 patients; (3) Thrombocytopenia: 7 patients; (4) Anemia: 7 patients; (5) Other, unspecified: 7 patients; (6) Neurologic disorders: 2 patients; (7) Cardiovascular events: 2 patients; (8) Secondary neoplasms: 5 patients/events listed in axi-cel recipients; (9) Psychiatric disorders: 1 patient; (10) Immune-related events: 1 patient	Neurologic; Hematological/Immunological; Infectious; Cardiopulmonary and vascular; SMs; Other	Confirmed: >12–24 months after CAR-T therapy, reported separately for >12–18 and >18–24 months	7/172 (4.1%); cumulative incidence 4.4% at 12 months and 6.3% at 24 months	12 and 24 months	All infections: COVID-19 pneumonia (*n* = 3), *E. coli*/Achromobacter sepsis (*n* = 1), *P. aeruginosa* sepsis (*n* = 1), *P. aeruginosa*/*E. coli* pneumonia with persistent COVID-19 (*n* = 1), and S. pneumoniae pneumonia with persistent COVID-19 (*n* = 1)
Gazeau et al. [21]	Product comparison/domain groups: axicabtagene ciloleucel *n* = 319; tisagenlecleucel *n* = 144; lisocabtagene maraleucel *n* = 43; brexucabtagene autoleucel *n* = 30; investigational CD19 CAR-T *n* = 3. Total: 539.	(1) Therapy-related myeloid neoplasms (t-MDS/t-AML): 22/29 patients overall;	Infectious; SMs; Other	Confirmed: 22 t-MN diagnoses after 12 months	57 total NRM deaths; cumulative incidence 5.4%, 9.7%, 12.0%, and 14.0% at 1, 2, 3, and 4 years	1, 2, 3, and 4 years after CAR-T infusion	Infection *n* = 22 (39%); t-MN *n* = 19 (33%); other causes *n* = 16 (28%)
Jagannath et al. [22]	Single product: ciltacabtagene autoleucel; *n* = 97.	(1) Second primary malignancies (solid tumors): 2 events (2) Neurologic adverse events, unspecified: 2 events; (3) Grade ≥ 3 infections: 4 events	Neurologic; Infectious; SMs	≥60 months in patients remaining progression-free for ≥60 months; (Long-term survivor status does not establish first onset at or after 12 months)	47 deaths overall; 17 deaths without progressive disease noted in disposition, but causes not reported as NRM in extracted text	Not reported as NRM	Not reported for NRM
Shah et al. [23]	Single product: brexucabtagene autoleucel/KTE-X19; *n* = 78.	(1) Graft-versus-host disease: 3 events; (2) Hypoxia with acute respiratory distress syndrome and hypotension: 1 event; (3) Infection, unspecified: 1 event; (4) Cardiopulmonary arrest: 1 event; (5) Intracranial hemorrhage related to relapsed ALL with thrombocytopenia: 1 event; (6) Missing cause: 1 event	Hematological/Immunological; Infectious; Cardiopulmonary and vascular; Other	Approx. 18.2, 21.9, 25.4, 25.6, 38.9, 38.9, and 47.0 months after infusion (The reported dates were death dates rather than first onset dates)	36-month non-PD mortality 19% (*n* = 15); 17 non-PD deaths at data cutoff	36 months and data cutoff (median follow-up 41.6 months)	Most common non-PD causes included GVHD (*n* = 3), sepsis (*n* = 2), pneumonia (*n* = 2); other listed causes included hypoxia/ARDS, hemorrhagic shock, herpes simplex viremia, infection plus GVHD, cardiopulmonary arrest, pulmonary GVHD, intracranial hemorrhage related to relapsed ALL, and missing cause
Abramson et al. [24]	Single product: lisocabtagene maraleucel; *n* = 345 leukapheresed; *n* = 270 treated.	(1) Nonmelanoma skin cancer: 9 reported events; (2) Myelodysplastic syndrome: 8 reported events; (3) Acute myeloid leukemia: 2 reported events	SMs	Confirmed: AML at approximately 18 months (1 event); Additional posttreatment-emergent SM entries from day 91 through 24 months	133 deaths after liso-cel; 110 due to disease progression; 11 deaths due to AEs (4%); 1 posttreatment-emergent grade 5 death of unknown primary cause after retreatment	Not reported as NRM	AE death causes included PML and septic shock (*n* = 2 each), pulmonary hemorrhage, multiple organ dysfunction, diffuse alveolar damage, leukoencephalopathy, cardiomyopathy, MDS, and AML (*n* = 1 each); no COVID-19 deaths
Berning et al. [25]	Two products: axicabtagene ciloleucel *n* = 113 (65.7%); tisagenlecleucel *n* = 59 (34.3%). Total: 172.	(1) Bacterial sepsis: 1 late fatal infection case (2) COVID-19: 1 late fatal infection case	Infectious	Bacterial sepsis approx. 18.9 months; COVID-19 approx. 21.8 months (The reported dates were death dates rather than first onset dates)	30/172 (17.4%) NRM overall; 12 early and 18 late; 12-month NRM 14.1% (<70) vs. 22.6% (≥70)	1, 3, 6, 12, and 24 months; early vs. late NRM also reported	Infections grade 5 *n* = 18; CRS/ICANS grade 5 *n* = 4; embolism *n* = 3; other reasons *n* = 5
Jain et al. [16]	Single product: axicabtagene ciloleucel; *n* = 298 leukapheresed; *n* = 275 treated.	(1) Infections: 21 NRM deaths total; 13 occurred at ≥12 months (6 unclassified, 5 pneumonia, 4 bacterial sepsis, 2 COVID-19, 2 candidemia, 1 candidemia with concomitant Pneumocystis jirovecii pneumonia, 1 viral encephalitis); (2) SMs: 15 NRM deaths total; 9 occurred at ≥12 months (11 MDS, 2 AML, 1 CML, 1 mast cell leukemia); (3) Other malignancies: 9 deaths total (anal cancer, prostate cancer, endometrial cancer, lung cancer, metastatic Merkel cell carcinoma, mesothelioma, histiocytic sarcoma, B-cell ALL, angioimmunoblastic T-cell lymphoma); (4) Suicide: 1 death	Neurologic; Infectious; SMs	Confirmed: 9 severe infection events during months 12–24; 9 t-MN diagnoses at or after 12 months; and 1 AITL diagnosed after month 17 (Other death timing and untimed entries remained onset uncertain)	40 NRM deaths; 5-year NRM 16.2%	5-year follow-up; causes reported by year after infusion	Infection *n* = 21; SM *n* = 9; early CAR-T toxicity *n* = 3; suicide *n* = 1; unknown in remission *n* = 6
Locke et al. [26]	Single product: axicabtagene ciloleucel; *n* = 38.	(1) COVID-19: 1 event; (2) Myelodysplastic syndrome: 1 event.	Infectious; SMs	Confirmed: 24–48 months after CAR-T therapy	At 24 months: 19 deaths, including 15 after disease progression and 4 AE-related deaths; after the 24-month cutoff, one additional death occurred after MDS progressed to grade 5	Not reported as NRM	AE-related deaths included bacteremia, necrotizing pneumonia, renal failure after subsequent alloSCT, and axi-cel-related cerebral edema; later death due to grade 5 MDS
Lorenc et al. [27]	Multiple products: axicabtagene ciloleucel *n* = 188; tisagenlecleucel *n* = 76; lisocabtagene maraleucel *n* = 73; brexucabtagene autoleucel *n* = 18. Total: 355.	(1) Small cell lung cancer: 2 events; (2) Melanoma: 1 event; (3) Appendix adenocarcinoma: 1 event; (4) Myelodysplastic syndrome: 5 events; (5) Skin squamous cell carcinoma: 3 events; (6) Thyroid papillary carcinoma: 1 event; (7) Bladder carcinoma: 1 event; (8) Non-small cell lung cancer: 1 event	SMs	Confirmed: Approx. 13–41 months after CAR-T therapy, depending on malignancy type	129 deaths total; 103 recurrent/progressive lymphoma; 4 subsequent malignancy; 15 infection; 1 intestinal perforation; 1 organ failure; 1 intracranial hemorrhage; 1 portal vein thrombosis; 3 unknowns	Not reported as NRM	Non-progression causes include subsequent malignancy, infection, organ failure, intracranial hemorrhage, portal vein thrombosis, and unknown
Neelapu et al. [28]	Single product: axicabtagene ciloleucel; *n* = 159 enrolled; *n* = 152 treated.	(1) COVID-19 with pneumonia: 1 event; (2) Pyrexia: 1 event; (3) Myelodysplastic syndrome: 1 event; (4) Febrile neutropenia: 1 event; (5) Pneumonia: 1 event	Hematological/Immunological; Infectious; General or systemic condition; SMs	Five events reported after the 18-month analysis cutoff; individual post-infusion onset times were not reported	For FL: competing risks in lymphoma-specific PFS *n* = 12 (9%), 36-month cumulative incidence 10.7%; lymphoma-specific OS competing risks *n* = 16 (13%), 36-month cumulative incidence about 12%	36 months for competing-risk analyses	Deaths due to other reasons included infections/COVID-19, second primary malignancy, GVHD complications, unknown causes, and other AEs in Table S2
Czapka et al. [29]	Multiple products: axicabtagene ciloleucel *n* = 44; tisagenlecleucel *n* = 26; brexucabtagene autoleucel *n* = 2; lisocabtagene maraleucel *n* = 1. Total: 73.	(1) Respiratory viral infection: 8 events; (2) Pneumonia: 6 events; (3) Urinary tract infection: count unclear; (4) Shingles: count unclear; (5) Bacterial sinusitis: count unclear; (6) Herpes zoster: count unclear	Infectious	Confirmed: 12–24 months after CAR-T infusion	Not reported	Not reported	Not reported
Neelapu et al. [30]	Single product: axicabtagene ciloleucel; *n* = 111 enrolled; *n* = 101 treated.	(1) Myelodysplastic syndrome: 1 event; (2) Unknown other after-effects/death category: 4 events	SMs; Other	MDS-related death after the 2-year analysis; MDS diagnosis onset was not reported. One unknown death cause was excluded as a complication	59 deaths; 45 progressive diseases; 4 AE; 1 secondary malignancy; 9 other	Not reported as NRM	Death causes include AE, secondary malignancy, infection, cardiac arrest, pulmonary nocardiosis, sepsis, allogeneic transplantation complications, and unknown
Westin et al. [31]	Treatment comparison: axicabtagene ciloleucel arm *n* = 180 assigned (*n* = 170 treated) vs. standard care *n* = 179 assigned. Total randomized: 359.	(1) Hepatitis B reactivation: 1 event in the axi-cel arm	Infectious	Approx. 12 months after axi-cel infusion (HBV reactivation after late treatment discontinuation; exact onset was not reported)	Safety population deaths: axi-cel 74, standard care 91; progressive disease deaths 51 vs. 71; fatal AEs 8 vs. 2	Not reported as NRM	Axi-cel fatal AEs included COVID-19 (*n* = 2), sepsis (*n* = 2), HBV reactivation (*n* = 1), myocardial infarction (*n* = 1), pneumonia (*n* = 1), PML (*n* = 1); new/secondary cancer deaths *n* = 2
Little et al. [32]	Multiple products: axicabtagene ciloleucel *n* = 244; tisagenlecleucel *n* = 22; brexucabtagene autoleucel *n* = 8; lisocabtagene maraleucel *n* = 6. Total: 280.	(1) Pneumocystis jirovecii pneumonia: 2 late cases; 3 cases overall including one earlier case	Infectious	Confirmed: Late PJP at approx. 12.8 and 14.5 months; one additional overall PJP case at approx. 3.8 months	68 deaths (24%); majority (74%) related to primary disease	Not reported as NRM	One death due to septic shock in the setting of C. tropicalis fungemia; no other IFD primary death
Baird et al. [33]	Single product: axicabtagene ciloleucel; *n* = 41.	(1) Opportunistic fungal infection/Pneumocystis jirovecii pneumonia: 1 extracted late event	Infectious	Confirmed: Opportunistic infections reported up to 18 months; extracted late event approx. 12.8–13.8 months	NRM 2.4% (*n* = 1) at 1 year	1 year	Pneumocystis jirovecii pneumonia
Shah et al. [34]	Single product: brexucabtagene autoleucel/KTE-X19; *n* = 54 enrolled; *n* = 45 treated.	(1) Sepsis: 1 event	Infectious	Sepsis-related death approx. 19 months after CAR-T infusion; sepsis onset was not reported separately	26 treated patients died: 19 disease progression and 7 AEs	Not reported as NRM	AE deaths included 2 treatment-related early deaths; 5 unrelated AE deaths including sepsis (days 50 and 579), cerebrovascular accident, herpes simplex viremia, and bacteremia
Wudhikarn et al. [35]	Two products: axicabtagene ciloleucel *n* = 43 (71.7%); tisagenlecleucel *n* = 17 (28.3%). Total: 60.	(1) Hepatic/portal vein thrombosis with acute fulminant hepatic failure: 1 event	Hematological/Immunological	Approx. 12 months after CAR-T infusion (Fatal hepatic or portal vein thrombosis in 1-year NRM reporting; onset date was not reported)	Two patients died in remission; 1-year NRM 1.7% (95% CI, 0.1–8.0%)	1 year	Extensive hepatic/portal vein thrombosis at 1 year
Locke et al. [36]	Single product: axicabtagene ciloleucel; *n* = 119 enrolled; *n* = 108 treated.	(1) Lung infection: 1 event; (2) Bacteremia: 1 patient with 2 episodes; (3) Myelodysplastic syndrome: 1 event	Infectious; SMs	Confirmed: Lung infection at 19.3 months; bacteremia at 15.5 and 20.7 months; MDS at 18.9 months	54/108 treated patients died; 50 from PD; 4 AE-related deaths occurred earlier in study; no new treatment-related deaths during additional follow-up	Not reported as NRM	Late serious AEs were lung infection, bacteremia, and MDS; none judged treatment-related; deaths not linked to these late AEs in extracted text

Notes: Denominators are reported as enrolled, treated, safety-set, or landmark populations and are not uniform 12-month at-risk denominators. No product-specific results are reported unless stated otherwise. Abbreviations: AE, adverse event; ALL, acute lymphoblastic leukemia; alloSCT, allogeneic stem cell transplantation; AML, acute myeloid leukemia; ARDS, acute respiratory distress syndrome; axi-cel, axicabtagene ciloleucel; CAR-T, chimeric antigen receptor T-cell; CD19, cluster of differentiation 19; CI, confidence interval; CML, chronic myeloid leukemia; COVID-19, coronavirus disease 2019; CRS, cytokine release syndrome; FL, follicular lymphoma; GVHD, graft-versus-host disease; HBV, hepatitis B virus; ICANS, immune effector cell-associated neurotoxicity syndrome; IFD, invasive fungal disease; KTE-X19, brexucabtagene autoleucel; LC, late complication; liso-cel, lisocabtagene maraleucel; MDS, myelodysplastic syndrome; NRM, non-relapse mortality; OS, overall survival; PD, progressive disease; PFS, progression-free survival; PJP, Pneumocystis jirovecii pneumonia; PML, progressive multifocal leukoencephalopathy; SMs, secondary malignancies; t-AML, therapy-related acute myeloid leukemia; t-MDS, therapy-related myelodysplastic syndrome; t-MN, therapy-related myeloid neoplasm.

## Data Availability

The data supporting the findings of this review are included in the article and Supplementary Materials. Further inquiries can be directed to the corresponding author.

## Supplementary Materials

The following supporting information can be downloaded at: https://www.mdpi.com/article/10.3390/curroncol33080461/s1, Table S1: Applied search string per database; Table S2: Overview of late complications reported, sorted by cluster; Table S3: Joanna Briggs Institute (JBI) Critical Appraisal Checklist for cohort studies.
