# Supplementary material for: Late Complications After European Medicines Agency-Approved Chimeric Antigen Receptor T-Cell Therapy in Hematological Malignancies: A Scoping Review
Source: Curr Oncol. 2026 Aug 1;33(8):461. doi: 10.3390/curroncol33080461 (PMC13511656; doi:10.3390/curroncol33080461)
Supplement: Supplementary file 1 [file curroncol-33-00461-s001.zip › curroncol-4416331-supplementary.pdf]

Supplementary Materials

Table S1: Applied search string per database.

	CAR-T terms	Temporal terms
PubMed	(<br>"Receptors, Chimeric Antigen"[Mesh] OR<br>"CAR-T*"[tiab] OR "CART"[tiab] OR "CAR T"[tiab] OR "CART therapy"[tiab] OR<br>"chimeric antigen receptor T-cell"[tiab] OR<br>"CAR modified T cells"[tiab] OR<br>"CAR T-cell therapy"[tiab] OR "engineered T cells"[tiab] OR<br>"genetically modified T lymphocytes"[tiab] OR<br>"adoptive T cell therapy"[tiab]<br>OR<br>Kymriah OR tisagenlecleucel OR<br>Yescarta OR axicabtagene OR axicabtagene ciloleucel OR<br>Tecartus OR brexucabtagene OR brexucabtagene autoleucel OR<br>Breyanzi OR lisocabtagene OR lisocabtagene maraleucel OR<br>Abecma OR idecabtagene OR idecabtagene vicleucel OR<br>Carvykti OR ciltacabtagene OR ciltacabtagene autoleucel<br>)	AND (<br>"Long-Term Adverse Effects"[MeSH] OR<br>"long-term"[tiab] OR<br>"long-term toxicity"[tiab] OR<br>"long-term complication*"[tiab] OR<br>"long-term adverse effect*"[tiab] OR<br>"long-term outcome*"[tiab] OR<br>"long-term follow-up"[tiab] OR<br>"late effect*"[tiab] OR<br>"late event*"[tiab] OR<br>"late toxicity"[tiab] OR<br>"late onset toxicity"[tiab] OR<br>"after treatment"[tiab] OR<br>"chronic side effect*"[tiab] OR<br>"delayed adverse event*"[tiab] OR<br>"follow-up"[tiab] OR<br>"outcome*"[tiab] OR<br>"Patient Reported Outcome"[tiab] OR<br>"persistent symptom*"[tiab] OR<br>"12 month* follow-up"[tiab] OR<br>"survivor*"[tiab] OR<br>"survivorship"[tiab]<br>)
Cochrane	(<br>CAR-T* OR CART OR "CAR T" OR "CART therapy" OR<br>"chimeric antigen receptor T-cell" OR "CAR modified T cells" OR<br>"CAR T-cell therapy" OR "engineered T cells" OR<br>"genetically modified T lymphocytes" OR<br>"adoptive T cell therapy" OR<br>Kymriah OR tisagenlecleucel OR<br>Yescarta OR axicabtagene OR "axicabtagene ciloleucel" OR<br>Tecartus OR brexucabtagene OR<br>"brexucabtagene autoleucel" OR<br>Breyanzi OR lisocabtagene OR "lisocabtagene maraleucel" OR<br>Abecma OR idecabtagene OR "idecabtagene vicleucel" OR<br>Carvykti OR ciltacabtagene OR "ciltacabtagene autoleucel"<br>)	AND (<br>"long-term" OR long-term NEXT toxicity OR<br>long-term NEXT complication* OR<br>long-term NEXT adverse NEXT effect* OR<br>long-term NEXT outcome* OR<br>long-term NEXT follow-up OR<br>late NEXT effect* OR<br>late NEXT event* OR<br>"late toxicity" OR<br>late NEXT onset NEXT toxicity OR<br>"after treatment" OR<br>chronic NEXT side NEXT effect* OR<br>delayed NEXT adverse NEXT event* OR<br>follow-up OR<br>outcome* OR<br>"Patient Reported Outcome" OR<br>persistent NEXT symptom* OR<br>"12 month" NEXT follow-up OR<br>survivor* OR survivorship

	CAR-T terms	Temporal terms
		)
Web of Science	(TS=<br><br>((“CAR-T*” OR “CART” OR “CAR T” OR “CART therapy” OR “chimeric antigen receptor T-cell” OR “CAR modified T cells” OR “CAR T-cell therapy” OR “engineered T cells” OR “genetically modified T lymphocytes” OR “adoptive T cell therapy” OR “Kymriah” OR “tisagenlecleucel” OR “Yescarta” OR “axicabtagene” OR “axicabtagene ciloleucel” OR “Tecartus” OR “brexucabtagene” OR “brexucabtagene autoleucel” OR “Breyanzi” OR “lisocabtagene” OR “lisocabtagene maraleucel” OR “Abecma” OR “idecabtagene” OR “idecabtagene vicleucel” OR “Carvykti” OR “ciltacabtagene” OR “ciltacabtagene autoleucel”)))	AND TS=<br><br>((“long-term” OR “long-term toxicity” OR “long-term complication*” OR “long-term adverse effect*” OR “long-term outcome*” OR “long-term follow-up” OR “late effect*” OR “late event*” OR “late toxicity” OR “late onset toxicity” OR “after treatment” OR “chronic side effect*” OR “delayed adverse event*” OR “follow-up” OR “outcome*” OR “Patient Reported Outcome” OR “persistent symptom*” OR “12 month* follow-up” OR “survivor*” OR “survivorship”))

Table S2: Overview of late complications reported, sorted by cluster.

Cluster	Types of late complications	Number of types	Number of studies
Neurologic	Neurologic disorder (unspecified), Psychiatric disorders (unspecified), Suicide, Unspecified cause	4	3
Hematological/immunological	Febrile neutropenia, Graft-versus-host disease, Hepatic vein thrombosis, Neutropenia, Thrombocytopenia, Anemia	6	4
Infectious	Bacteremia, Bacterial sinusitis, Candidemia, COVID-19, Encephalitis (viral), Hepatitis B reactivation, Herpes zoster, Infection (fungal, unspecified), Infection (Shingles), Infection (unspecified), Lung infection (unspecified), Pneumocystis jirovecii pneumonia, Pneumonia, Respiratory virus infection, Urinary tract infection, Sepsis (bacterial)	16	14
Cardiopulmonary and vascular	Cardiopulmonary arrest, Cardiovascular event (unspecified), Hypoxia with acute respiratory distress syndrome and accompanying hypotension, Intracranial hemorrhage (due to ALL-related thrombocytopenia)	4	2

Cluster	Types of late complications	Number of types	Number of studies
General or systemic condition	Pyrexia	1	1
Secondary malignancies	Acute lymphatic leukemia, Acute myeloid leukemia, Anal cancer (unspecified), Angioimmunoblastic T-cell lymphoma, Chronic myeloid leukemia, Appendix adenocarcinoma, Basal cell carcinoma, Bladder carcinoma, Endometrial cancer, Histiocytic sarcoma, Mast cell leukemia, Melanoma, Merkel cell carcinoma, Mesothelioma, Myeloid neoplasms (therapy-related), Myelodysplastic syndrome, Non-melanoma skin cancer, Non-small cell lung cancer, Prostate cancer, Second primary malignancies (Solid tumor, unspecified), Cutaneous squamous cell carcinoma, Small cell lung cancer, Thyroid papillary carcinoma, Unspecified cause	24	10
Other	Missing causes (NRM-related), Unspecified cause	2	4

Table S3: Joanna Briggs Institute (JBI) Critical Appraisal Checklist for cohort studies

Item	Question
Q1	Were the two groups similar and recruited from the same population?
Q2	Were the exposures measured similarly to assign people to both exposed and unexposed groups?
Q3	Was the exposure measured in a valid and reliable way?
Q4	Were confounding factors identified?
Q5	Were strategies to deal with confounding factors stated?
Q6	Were the groups/participants free of the outcome at the start of the study?
Q7	Were the outcomes measured in a valid and reliable way?
Q8	Was the follow-up time reported and sufficient to be long enough for outcomes to occur?
Q9	Was follow-up complete, and if not, were the reasons to loss to follow up described and explored?
Q10	Were strategies to address incomplete follow-up utilized?
Q11	Was appropriate statistical analysis used?
